# Feasibility of Laparoscopic Sleeve Gastrectomy for Patients with Obesity and Disorders of Intellectual Development: a Single Institutional Experience

**DOI:** 10.1007/s11695-023-06543-0

**Published:** 2023-03-21

**Authors:** Kotaro Wakamatsu, Takashi Oshiro, Natsumi Kitahara, Yuuki Moriyama, Taiki Nabekura, Kozue Hashi, Karin Hayashi, Atsuhito Saiki, Shinichi Okazumi

**Affiliations:** 1 Department of Surgery, Toho University Sakura Medical Center, 564-1 Shimoshizu Sakura, Chiba, 285-8741 Japan; 2 Department of Neuropsychiatry, Toho University Sakura Medical Center, 564-1 Shimoshizu Sakura, Chiba, 285-8741 Japan; 3 Department of Psychiatry, Toho University Sakura Medical Center, 564-1 Shimoshizu Sakura, Chiba, 285-8741 Japan; 4 Center of Diabetes, Endocrine and Metabolism, Toho University Sakura Medical Center, 564-1 Shimoshizu Sakura, Chiba, 285-8741 Japan

**Keywords:** Laparoscopic sleeve gastrectomy, LSG, Disorders of intellectual development

## Abstract

**Background:**

Owing to their difficulty following clinical advice for procedural safety and ideal surgical outcomes, bariatric and metabolic surgery (BMS) for patients with disorders of intellectual development (DID) is concerning. Studies reporting the feasibility of BMS for this population remain scarce. This study aims to clarify the feasibility of laparoscopic sleeve gastrectomy (LSG) for patients with clinically severe obesity and DID.

**Methods:**

A retrospective analysis of a single institutional prospective database collected from 2010 to 2022 was performed. The Wechsler Adult Intelligence Scale (WAIS) was used to measure intellectual ability before LSG. A multidisciplinary team approach was implemented to give special support and care to patients with DID. Patients were categorized into groups according to their WAIS scores. LSG outcomes were statistically compared between the DID and average intellectual ability groups.

**Results:**

Using the WAIS to measure intellectual ability among patients who underwent LSG, we identified 14 patients with DID (IQ score: < 69, mean IQ: 63.4) and 71 with average intellectual ability (IQ score: 90–109, mean IQ: 98.9). Operative outcomes were comparable between the groups as follows: operation time (DID: 163 ± 41 min, average intelligence: 162 ± 30 min), hospital stay (DID: 4 [4–5] days, average intelligence: 5 [4–6] days), and total comorbidities (DID: 7.1%, average intelligence: 8.4%). No reoperations were performed, and no mortalities were observed.

**Conclusions:**

With medical and social support and care, performing LSG on patients with clinically severe obesity and DID is safe, with good short-term results.

**Graphical abstract:**

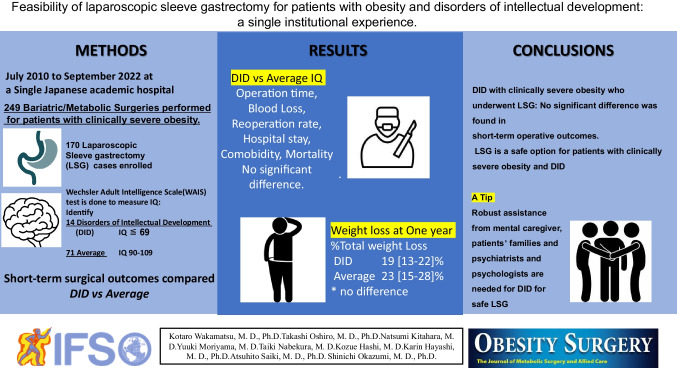

## Introduction

Although the incidence of severe obesity is reportedly higher among individuals with disorders of intellectual development (DID) [1, 2], there is no clear surgical contraindication or guideline for bariatric and metabolic surgery (BMS) in this population [3]. Patients with DID may experience difficulty following nutritional guidelines and face social barriers when performing physical activities, leading to greater difficulty in reducing excessive weight and sustaining a healthy weight than people with average intellect. Most case studies have reported BMS outcomes among patients with Prader–Willi syndrome [4]; however, studies adequately supporting the safety of BMS among the DID population are still lacking. Herein, we retrospectively analyzed the feasibility of laparoscopic sleeve gastrectomy (LSG) in a DID population compared to that in patients with average intellectual ability.

## Materials and Methods

### Participants

**Fig. 1:**
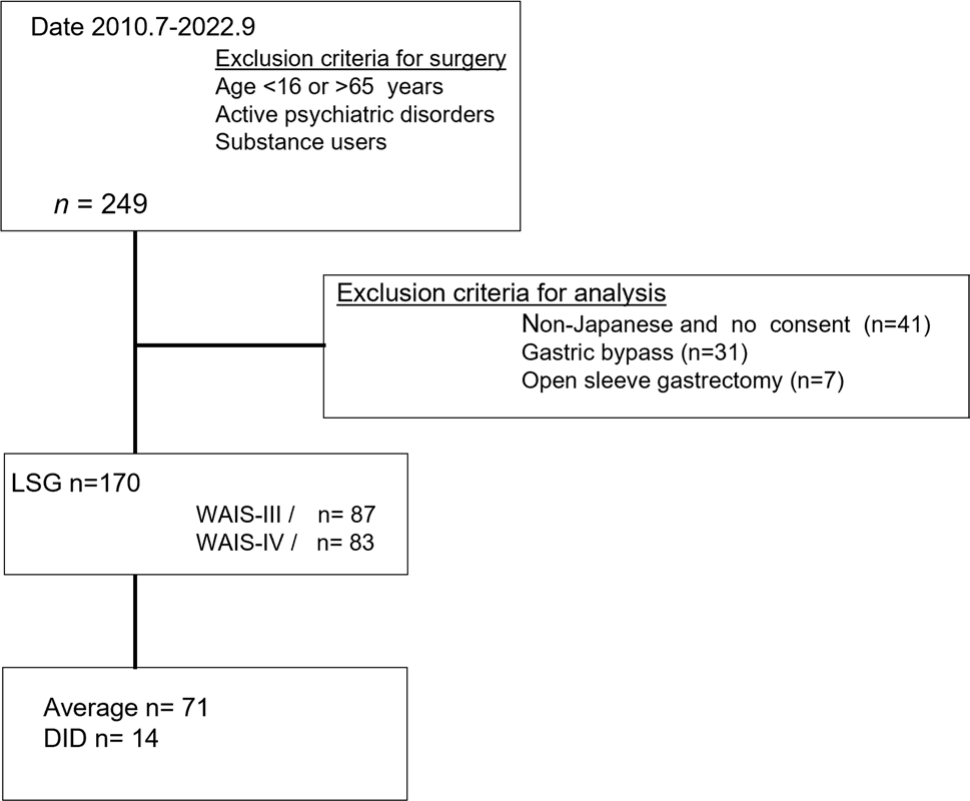
Patient demographics

This was a retrospective analysis of a prospective database collected from July 2010 to September 2022 at a Japanese academic hospital; 249 patients were identified. Patients who did not consent to assessment with the Wechsler Adult Intelligence Scale (WAIS) tests, underwent bypass or open sleeve gastrectomy, were non-Japanese, or had missing data were excluded from the analysis. Finally, 170 patients with complete data who underwent LSG for clinically severe obesity were included (Fig. 1).

Bariatric and metabolic surgery (BMS) was indicated according to the criteria established in 2005 by the Asia Pacific Bariatric Surgery Group Consensus meeting [3]. All patients were provided with up-to-date detailed information regarding available BMS options, including potential complications and nutritional requirements after surgery.

### Review Board for Obesity Treatment

A multidisciplinary approach to obesity treatment was used in our hospital. Patients were admitted to the Center of Diabetes, Endocrine, and Metabolism department for approximately 2 weeks before BMS, during which internal physicians and dietitians performed routine physical checkups to exclude secondary causes of obesity and provided physical activity and nutritional advice. Pharmacotherapy for obesity-related comorbidities was standardized, and consultations with psychiatrists and psychologists were co-initiated to rule out and support any psychosocial problems and mental disorders. Finally, eligibility for BMS was discussed in a conference based on the joint consensus statement from the Japanese Society for Treatment of Obesity, the Japan Diabetes Society, and the Japan Society for the Study of Obesity [5]. Patients with confirmed eligibility were referred to surgeons as candidates for LSG. Following discharge, patients periodically consulted with physicians, dieticians, psychiatrists, and psychologists. Of note, a total of 14 patients were suspended from proceeding to BMS by this board. The reasons for the suspensions were as follows: four patients had uncontrolled depression disorder, one had Prader–Willi syndrome with an uncontrolled eating disorder, one had an unstable status after brain surgery, three were unable to adhere to nutritional management, and five were unwilling to take BMS as recommended by physicians. One patient died from suicide during regular visits, and one patient underwent BMS at another hospital.

### Measurements

Obesity-related disorders were diagnosed by specialists from our institution. Liver diseases included nonalcoholic fatty liver disease, nonalcoholic steatohepatitis, and liver cirrhosis. Mental diseases included depression, bipolar disorder, and schizophrenia.

Operation time, blood loss, reoperation rate, surgical comorbidities (Clavien–Dindo classification grade ≥ II), mortality, and duration of hospital stay were recorded as operational outcomes, and patients’ weight was measured at every hospital visit. The visceral fat area (VFA) and subcutaneous fat area (SFA) were calculated using SYNAPSE VINCENT (Fujifilm) in the L3 region.

### Evaluation of WAIS

Under the supervision of trained psychiatrists or psychologists, the Japanese version of the WAIS test was completed by patients who underwent BMS surgery with consent; this took approximately 60–120 min to complete. The test was paper-based comprising four indexes to assess broad aspects of intellectual ability, including verbal comprehension, perceptual reasoning, working memory, and processing speed. The total score of the four indexes represented full-scale IQ (FSIQ). During this study, the WAIS was updated from version III to IV, which was released in 2018 (the test is regularly updated to maintain a proper score distribution). Patients with an FSIQ of ≦ 69 were considered to have DID, while patients with an FSIQ of 90–109 were considered to have an average IQ.

### Surgical Procedure

The LSG method was previously described [6]. First, a laparoscopic trocar was inserted using an optical method. After establishing pneumoperitoneum at 10–15 mmHg, four additional laparoscopic trocars were placed in the upper abdomen and a liver retractor was also inserted. After the division of the short gastric vessels and complete mobilization of the fundus with exposure of the left crus, the stomach was vertically transected using multiple applications of a linear stapler over a 36-French orogastric bougie. The first staple was applied 5 cm from the pyloric ring. The staple line was oversewn with a running 2–0 nonabsorbable suture, keeping the bougie in place. The sleeved stomach was fixed to the mesocolon using two simple 2–0 nonabsorbable sutures to prevent axial rotation of the sleeved stomach.

Postoperatively, patients spent one night in the intensive care unit for intensive vital monitoring. Patients were usually discharged on postoperative day 4 after receiving dietary instructions from dieticians, and proton pump inhibitors were continued for at least 6 months. Follow-up visits were universally scheduled every month until 6, 9, 12, 18, and 24 months; they were then scheduled annually. Laboratory evaluations and weight measurements were conducted at every visit.

### Statistical Analysis

Continuous variables are shown as the mean and standard deviation (SD) or median and interquartile range, while categorical variables are shown as the number and percentage. Parametric data were compared using the paired Student’s *t-*test, while nonparametric data were analyzed using the Mann–Whitney U test. WAIS test score distributions were analyzed using the Kolmogorov–Smirnov test. All statistical analyses were conducted using R software version 3.2.3 (R Foundation for Statistical Computing, Vienna, Austria). The test was two-sided, and a 5% significance level was used.

## Results

Of the 249 patients listed in our database, 41 who did not consent to taking WAIS tests were not Japanese natives or had missing data; seven who underwent open sleeve gastrectomy and 31 who underwent gastric bypass were excluded. A total of 170 patients were enrolled in this study. WAIS-III and WAIS-IV tests were completed by 87 and 83 patients, respectively. The total IQ score was lower for the WAIS-IV than for the WAIS-III (90.0 ± 15.6 vs. 95.6 ± 16.2, *p* = 0.02). Seven patients were identified as having DID on the WAIS III and seven on the WAIS IV. Overall, 14 (8.2%) patients with DID were compared with 71 (41.8%) patients with average IQ (WAIS-III/IV, *n* = 39/32; Table 1).

**Table 1:** WAIS-III/IV score. Distributions of WAIS-III and WAIS-IV scores are shown

	WAIS-III	WAIS-IV	*p*-value
*n*	87 (51%)	83 (49%)	
*FSIQ*	95.6 ± 16.2	90.0 ± 15.6	0.02*
*IQ score*			
≤ 69	7 (8%)	7 (8.4%)	
70–79	7 (8%)	14 (17%)	
80–89	17 (20%)	20 (24%)	
90–109	39 (45%)	32 (39%)	
110–119	10 (11%)	7 (8.4%)	
120–129	6 (6.8%)	2 (2.4%)	
≥ 130	1 (1.2%)	1 (1.2%)	

A *p*-value < 0.05 is considered a statistically significant difference (*)

IQ score ranks: ≦ 65, disorders of intellectual development; 70–79, borderline; 80–89, low-average; 90–109, average; 110–119, high average; 120–129, superior; ≧ 130, very superior

*WAIS*, Wechsler Adult Intelligence Scale; *FSIQ*, full-scale intelligence quotient

Patients with DID had significantly lower total IQ scores and IQ subscores than patients with average IQ ([FSIQ] DID: 63.4 ± 4.6, average IQ: 98.9 ± 5.3; [verbal comprehension] DID: 70.4 ± 10.1, average IQ: 99.7 ± 7.2; [perceptual reasoning] DID: 70.8 ± 9.1, average IQ: 99.7 ± 10.7; [working memory] DID: 68.7 ± 11.6, average IQ: 97.6 ± 10.6; [processing speed] DID: 72.6, average IQ: 93.1 ± 12.1; all *p* < 0.05). Demographic data were compared between the DID and average IQ groups; age, sex, and BMI were comparable ([age] DID: 43.0 ± 10.9 years, average IQ: 45.1 ± 9.8 years, *p* = 0.47; [female sex] DID: 57%, average IQ: 42%, *p* = 0.47; [BMI] DID: 44.7 ± 7.8 kg/m^2^, average IQ: 45.6 ± 10.1 kg/m^2^, *p* = 0.79). Although the SFAs were comparable, VFA was significantly lower in DID group than in the average IQ group ([SFA] DID: 491 [355–722] cm^2^, average IQ: 483 [366–751] cm^2^, *p* = 0.82; [VFA] DID: 172 [139–213] cm^2^, average IQ: 232 [168–303] cm^2^, *p* = 0.02). No significant difference was observed regarding the frequency of obesity-related comorbidities (Table 2).

**Table 2:** Clinical characteristics of patients with disorders of intellectual development. Clinical characteristics were compared between patients with DID and those with average intellectual capabilities and clinically severe obesity

	DID	Average	*p*-value
*n*	14	71	
FSIQ score	63.4 ± 4.6	98.9 ± 5.3	< 0.05
VC	70.4 ± 10.1	99.7 ± 7.2	< 0.05
PR	70.8 ± 9.1	99.7 ± 10.7	< 0.05
WM	68.7 ± 11.6	97.6 ± 10.6	< 0.05
PS	72.6 ± 10.1	93.1 ± 12.1	< 0.05
*Demographic data*			
Age	43.0 ± 10.9	45.1 ± 9.8	0.47
Female/male	8 (57%)/6 (43%)	30 (42%)/41 (58%)	0.47
BMI (kg/m^2^)	44.7 ± 7.8	45.6 ± 10.1	0.79
VFA (cm^2^)	172 [139–213]	232 [168–303]	0.02*
SFA (cm^2^)	491 [355–722]	483 [366–751]	0.82
*Obesity-related comorbidities*			
Hypertension	6 (43%)	48 (68%)	0.15
T2DM	6 (43%)	44 (62%)	0.24
Dyslipidemia	10 (71%)	59 (82%)	0.51
SAS	7(50%)	35 (49%)	0.22
GERD	2 (14%)	7 (9.8%)	1
Liver diseases	13 (93%)	62 (87%)	1
CKD	1 (7%)	7 (9.8%)	0.82
Hyperuricemia	5 (36%)	35 (49%)	0.64
CVD	0 (0%)	7(9.8%)	0.48
Mental disorders	3 (21%)	20 (28%)	0.17
Depression	0	3	
Bipolar disorder	1	11	
Schizophrenia	2	1	
Anxiety disorder	0	4	
PTSD	0	1	

A *p*-value < 0.05 indicates a statistically significant difference (*)

*FSIQ*, full-scale intelligence quotient; *DID*, disorders of intellectual development; *VC*, verbal comprehension; *PR*, perceptual reasoning; *WM*, working memory; *PS*, processing speed; *BMI*, body mass index; *VFA*, visceral fat area; *SFA*, subcutaneous fat area; *T2DM*, type 2 diabetes mellitus; *SAS*, sleep apnea syndrome; *GERD*, gastroesophageal reflux disease; *CKD*, chronic kidney disease; *CVD*, cardiovascular disease; *PTSD*, post-traumatic stress syndrome

While patients in the DID group tended to require longer days from the first visit to LSG, the difference was not significant (639 [254–115] vs. 385 [247–799] days; *p* = 0.29). The lengths of hospital stay in both the surgery and internal medicine departments were comparable between groups ([surgery] DID: 4 [4–5.8] days, average IQ: 5 [4–6] days, *p* = 0.46; [internal medicine] DID: 14 [12.3–16] days, average IQ: 14 [4, 12–15] days, *p* = 0.599). All short-term surgical outcome parameters, including operation time (163 ± 41 min vs. 162 ± 30 min; *p* = 0.90), blood loss (5 [0–11]ml vs. 5 [0–10] ml; *p* = 0.63), and comorbidity rate (7.1% vs. 8.4%; *p* = 1.00) were comparable between the groups; neither mortalities nor reoperations were observed. The effect of LSG was measured by weight loss up to 1 year post-surgery, and no significant differences were observed between the DID and average intelligence groups (percentage of total body weight loss at 1 year: DID, 19% [13–22%]; *n* = 6/14 vs. average, 23% [15–28%]; *n* = 38/71; *p* = 0.34; Table 3).

**Table 3:** Surgical outcomes and weight loss. Short operative outcomes and weight loss after LSG were compared between patients with DID and those with average intellectual capabilities and clinically severe obesity

	DID	Average	*p*-value
Short operative outcomes			
Days to surgery (days)	639 [254–1158]	385 [247–799]	0.29
Hospital stay (internal medicine) (days)	14 [12–16]	14 [12–16]	0.59
Operation time (min)	163 ± 41	162 ± 30	0.9
Blood loss (ml)	5 [0–11]	5 [0–10]	0.63
Reoperation	0	0	N.D
Hospital stay (surgery) (days)	4 [4–5]	5 [4–6]	0.46
Total comorbidities, *n* (%)	1 (7.1%)	6 (8.4%)	1
Leakage	1	0	
Stenosis	0	3	
Bleeding	0	2	
AMI	0	1	
Mortality	0	0	
Total participant	14	71	
*Weight loss (% TWL): follow-up rate*			
3 months	16 [13–18]: *n* = 10/14	16 [12–18]: *n* = 53/71	0.72
6 months	15.3 [8.7–22]: *n* = 6/14	20.4 [15–24]: *n* = 47/71	0.27
12 months	19 [13–22]: *n* = 6/14	23 [15–28]: *n* = 38/71	0.34

*DID*, disorders of intellectual development; *AMI*, acute myocardial infarction; *% TWL*, percentage of total body weight loss; *N.D.*, not determined

## Discussion

The prevalence of obesity is higher among people with DID [1, 2]; this makes evaluation of the safety of BMS for patients with DID and clinically severe obesity a vital precaution. Preoperative psychopathology is associated with longer hospital stays, increased complications, and increased readmission after BMS [7–10]. Approximately 80% of bariatric programs consider severe DID (IQ < 50) as a definite contraindication, while approximately 50% believe that a mild-to-moderate DID (IQ = 50–70) is a definite contraindication for BMS [11]. Few BMS studies focusing on patients with clinically severe obesity and DID exist. Gibbons et al. [4] reviewed and summarized 16 case reports and series to investigate the safety of BMS for patients with DID. This review described 41 patients with DID, of whom 40 (97%) were diagnosed with Prader–Willi syndrome and suffered from DID and behavioral disorders. The short-term surgical outcomes of this study showed that 37.5% of patients experienced complications, and there were five fatalities (37%). In contrast to our results, the study by Gibbons et al. discouraged BMS in cases of DID due to its high mortality rate. It is noteworthy that the majority of procedures performed in their study were not sleeve gastrectomies; therefore, the feasibility and safety of LSG cannot be drawn from that of Gibbson’s report. With careful preoperative evaluation by our board for obesity treatment, our results showed no relationship between DID and poor short-term operative outcomes of LSG. This appears encouraging for the performance of BMS in patients with clinically severe obesity and DID. It should be noted that our study did not include patients with severe DID and that one patient with Prader–Willi syndrome had an uncontrolled eating disorder; therefore, the safety of BMS for DID in patients with lower IQ scores (< 50) and behavioral disorders should be clarified in the future.

Interestingly, patients with DID exhibited relatively low visceral fat accumulation. Visceral fat accumulation has been researched more intensively than subcutaneous fat accumulation and is strongly correlated with increased risk of metabolic dysfunction and cardiovascular disease [12–14]. In contrast, increased subcutaneous fat accumulation shows either no correlation or an inverse correlation with metabolic disease risk [15, 16]. Recent advances in adipocyte biology have revealed differing gene expression signatures and biological characteristics of adipocytes between visceral and subcutaneous fat accumulation. Moreover, 32 IQ trait-associated genes that impact human intelligence have recently been identified [17]. Similarly, our previous study revealed that early-onset obesity predominantly increases subcutaneous fat accumulation, suggesting that subcutaneous fat accumulation is correlated with genetic rather than environmental factors [18]. These findings may inspire future research to identify the genes responsible for adipocyte and neuron cell biology by comparing the genomes of patients with DID and those of patients with average IQ.

Coexisting behavioral disorders may negatively impact surgical outcomes [19]. It should be noted that the WAIS was only designed to measure broad cognition ability and not behavioral problems. If behavioral disorders (BD) are suspected, appropriate clinical techniques and questionnaires other than the WAIS must be applied for proper diagnosis and management. BD can be categorized into five disorders: attention deficient hyperactivity disorder, emotional behavioral disorder, oppositional defiant disorder, anxiety disorder, and obsessive–compulsive disorder. These behavioral disorders may overlap with each other and with DID and may be challenging to differentiate. The safety of BMS among patients with BD and DID has not been reported and remains a challenging problem to be addressed in future studies.

Psychosocial optimization is recommended before BMS to ensure satisfactory outcomes [20]. Preoperative psychosocial function can be assessed and adjusted by a broad range of qualified health caregivers. In our institution, internal physicians, dieticians, social workers, psychiatrists, and psychologists work as a team to optimize patients’ preoperative conditions. Our study revealed that patients with DID tend to require more time before LGS; this may reflect the complex condition of patients with DID as they require multiple hospital visits to access appropriate care for their mental disorders and they and their families must be educated about the importance of changing food habits. We did not indicate any clear psychosocial contraindications for BMS, but ongoing or uncontrolled mental disorders and current substance use were excluded; these conditions clearly require treatment of the mental disorder to be prioritized. It should be emphasized that the positive short-term postoperative outcomes for DID may have been due to the intensive support provided by the patients’ families and our psychiatrists and psychologists. In other words, satisfactory surgical outcomes for DID would not be guaranteed without diligent assistance from mental health caregivers.

The present study has several limitations. First, it only presents the feasibility of LSG performed on patients with both DID and clinically severe obesity. In addition, long-term weight changes with other obesity-related parameters and gastrointestinal quality were not studied; therefore, the long-term efficacy of LSG is unknown. Lastly, this was a single-center retrospective study with a relatively small number of cases; multicenter studies with adequate cases are required to draw a concrete conclusion.

## Conclusion

This present study compares the short-term surgical outcomes of patients with clinically severe obesity with and without DID. Our findings show that patients with clinically severe obesity and mild-to-moderate DID are feasible candidates for LSG. In addition, robust assistance from key persons and social support were vital to achieving these results.

## Data Availability

The datasets generated during and/or analysed during the current study are available from the corresponding author on reasonable request.
