# Correction: Feasibility of Laparoscopic Sleeve Gastrectomy for Patients with Obesity and Disorders of Intellectual Development: a Single Institutional Experience

**DOI:** 10.1007/s11695-023-06872-0

**Published:** 2023-09-30

**Authors:** Kotaro Wakamatsu, Takashi Oshiro, Natsumi Kitahara, Yuuki Moriyama, Taiki Nabekura, Kozue Hashi, Karin Hayashi, Atsuhito Saiki, Shinichi Okazumi

**Affiliations:** 1 Department of Surgery, Toho University Sakura Medical Center, 564-1 Shimoshizu Sakura, Chiba, 285-8741 Japan; 2 Department of Neuropsychiatry, Toho University Sakura Medical Center, 564-1 Shimoshizu Sakura, Chiba, 285-8741 Japan; 3 Department of Psychiatry, Toho University Sakura Medical Center, 564-1 Shimoshizu Sakura, Chiba, 285-8741 Japan; 4 Center of Diabetes, Endocrine and Metabolism, Toho University Sakura Medical Center, 564-1 Shimoshizu Sakura, Chiba, 285-8741 Japan

**Correction: Obesity Surgery (2023) 33:1327-1332**

**https://doi.org/10.1007/s11695-023-06543-0**

There is an error in the provided number within the Ethical Approval section of the article. The correct Ethical Approval number is IRB#S21023_S18061.

